# Soil respiration under climate change: prolonged summer drought offsets soil warming effects

**DOI:** 10.1111/j.1365-2486.2012.02696.x

**Published:** 2012-04-24

**Authors:** Andreas Schindlbacher, Steve Wunderlich, Werner Borken, Barbara Kitzler, Sophie Zechmeister-Boltenstern, Robert Jandl

**Affiliations:** 1 Department of Forest Ecology, Federal Research and Training Centre for Forests, Natural Hazards and Landscape – BFWSeckendorff-Gudent Weg 8, A-1131 Vienna, Austria; 2 Department of Soil Ecology, University of BayreuthDr. Hans Frisch Straβe 1-3, D-95448 Bayreuth, Germany; 3 Institute of Soil Research, University of Natural Resources and Applied Life Sciences – BOKUGregor Mendel Straβe 33, A-1180 Vienna, Austria

**Keywords:** 14C, drought, precipitation, roof, soil respiration, soil warming

## Abstract

Climate change may considerably impact the carbon (C) dynamics and C stocks of forest soils. To assess the combined effects of warming and reduced precipitation on soil CO_2_ efflux, we conducted a two-way factorial manipulation experiment (4 °C soil warming + throughfall exclusion) in a temperate spruce forest from 2008 until 2010. Soil was warmed by heating cables throughout the growing seasons. Soil drought was simulated by throughfall exclusions with three 100 m^2^ roofs during 25 days in July/August 2008 and 2009. Soil warming permanently increased the CO_2_ efflux from soil, whereas throughfall exclusion led to a sharp decrease in soil CO_2_ efflux (45% and 50% reduction during roof installation in 2008 and 2009, respectively). In 2008, CO_2_ efflux did not recover after natural rewetting and remained lowered until autumn. In 2009, CO_2_ efflux recovered shortly after rewetting, but relapsed again for several weeks. Drought offset the increase in soil CO_2_ efflux by warming in 2008 (growing season CO_2_ efflux in t C ha^−1^: control: 7.1 ± 1.0; warmed: 9.5 ± 1.7; warmed + roof: 7.4 ± 0.3; roof: 5.9 ± 0.4) and in 2009 (control: 7.6 ± 0.8; warmed + roof: 8.3 ± 1.0). Throughfall exclusion mainly affected the organic layer and the top 5 cm of the mineral soil. Radiocarbon data suggest that heterotrophic and autotrophic respiration were affected to the same extent by soil warming and drying. Microbial biomass in the mineral soil (0–5 cm) was not affected by the treatments. Our results suggest that warming causes significant C losses from the soil as long as precipitation patterns remain steady at our site. If summer droughts become more severe in the future, warming induced C losses will likely be offset by reduced soil CO_2_ efflux during and after summer drought.

## Introduction

Soils store huge quantities of organic C and the CO_2_ flux from soils to the atmosphere is one of the largest fluxes in the global C cycle. Additional C storage in soils could reduce atmospheric CO_2_ concentrations and mitigate climate change. On the other hand, loss of soil C to the atmosphere would further accelerate global climate change (IPCC, 2007). As the decomposition of soil organic matter (SOM) is a temperature-dependent process, there is concern that global warming increases the CO_2_ efflux from soil (Trumbore *et al*., 1996; Davidson *et al*., 2000). If increased soil CO_2_ efflux is not balanced by increased C uptake by plant biomass or soil, then warming could even turn whole biomes from current C sinks into C sources (Cox *et al*., 2000; Bellamy *et al*., 2005). Therefore, warming effects on soil C have received great attention. Even though there is progress in understanding the theoretical background behind the temperature response of soil CO_2_ efflux (reviewed in Davidson & Janssens, 2006; Conant *et al.,* 2011), the interaction with other environmental and physiological factors complicates a quantitative assessment of warming effects on soil CO_2_ efflux from forest ecosystems. Modeling approaches, which predicted high warming induced soil C loss throughout the next century(ies) (Cox *et al*., 2000; Jones *et al*., 2003; Friedlingstein *et al*., 2006), were somewhat contradicted by *in situ* warming studies where the detectable warming effect leveled-off after some years already (Rustad & Fernandez, 1998; Luo *et al*., 2001; Rustad *et al*., 2001; Strömgren, 2001; Melillo *et al*., 2002). The impact of warming on various SOM fractions and their contribution to the CO_2_ efflux makes a determination of the overall warming effect challenging (Davidson & Janssens, 2006; Risk *et al*., 2008; Karhu *et al*., 2010). Further complexity arises as plants and soil closely interact and as other environmental drivers such as precipitation influence the temperature response of SOM decomposition (Gu *et al*., 2004; Davidson *et al*., 2005; Högberg & Read, 2006). Precipitation and soil moisture play a crucial role in microbial growth and activity. As soil moisture decreases, substrate diffusion becomes limited and microbes physiologically adapt to lower water potentials (Schimel *et al*., 2007). This slows down biochemical process rates and hence soil respiration (Orchard & Cook, 1983; Stark & Firestone, 1995). In the context of climate change, shifts in soil moisture, if caused by warming itself or as a result of changing precipitation pattern, will influence future soil C dynamics. The way moisture and temperature affect the soil CO_2_ efflux can be deduced from seasonal soil respiration patterns during colder/warmer and wetter/dryer years (Davidson *et al*., 1998; Epron *et al*., 1999; Yuste *et al*., 2003; Lavigne *et al*., 2004). This information was also used to model the CO_2_ efflux, with relatively high accuracy (Reichstein *et al*., 2003; Gaumont-Guay *et al*., 2006; Yuste *et al*., 2007; Jassal *et al*., 2008). However, ecosystems adapt to regional climate conditions by long-term succession. If climate change rapidly shifts site conditions beyond current levels (thresholds), which is not unlikely, ecosystems may respond differently. How an ecosystem, or an ecosystem component (in our case a soil), responds to changing climate can be tested by multifactorial manipulations of single climate factors. Such manipulation experiments, however, are rare and to our knowledge have not yet been performed in forests.

In the present study, we investigated how a 4 °C increase in soil temperature, 25 days throughfall exclusion during July/August, and a combination of both affected the soil CO_2_ efflux in a temperate mountain forest in the European Alps. The experimental 4 °C temperature increase is within the range of IPCC scenarios for this century and summer droughts are predicted to occur more frequently in the study region (IPCC, 2007; Loibl *et al*., 2007). We hypothesized that (1) soil warming increases soil respiration, (2) summer throughfall exclusion decreases soil respiration, followed by a peak in soil respiration during natural rewetting. Since we excluded throughfall for only 25 days, while soil was warmed throughout the whole growing season, we further hypothesized (3) that throughfall exclusion compensates only a part of the warming effects on soil respiration.

## Materials and methods

### Site description

The study site is located at 910 m a.s.l. on a north-northeast slope of a mountain in the Northern Limestone Alps, Achenkirch, Austria (47° 34′ 50″ N; 11° 38′ 21″ E). The site is characterized by a cool humid climate. Snow-free period was from April/May to November/December. Mean annual air temperature and precipitation were 5.7 °C and 1480 mm (1987–2007), respectively. The 125-year-old forest is dominated by Norway spruce (*Picea abies*), with interspersed European beech (*Fagus sylvatica*) and silver fir (*Abies alba*). Understory mainly consists of beech regeneration. The soils are a mosaic of shallow Chromic Cambisols and Rendzic Leptosols. The bedrock is formed of dolomite. Soils are characterized by high carbonate content and near neutral pH. Mull is the dominant humus form and the depths of the litter + O-layer reach from 0 to 5 cm. A-horizons show a strong, small-scale variability in thickness reaching from 10 to 40 cm. Root density is highest in the O-, and A-horizons and few roots were found down to a depth of 60 cm. Organic C stocks were estimated to be ∼10 t ha^−1^ in the organic layer and ∼120 t ha^−1^ in the mineral soil (Schindlbacher *et al*., 2010).

### Experimental design and field measurements

In 2004, three experimental plots were randomly set on the site. The distance between individual plots was roughly 20 m ensuring similar slope and aspect of all plots. Each of the three plots consisted of a warmed, a disturbed-control, and a control subplot. At the disturbed-control plots, we buried a dummy cable to separate warming effects from initial disturbance effects following cable insertion. Subplots had a size of 2 × 2 m each. A 1 m wide buffer strip was kept between control and warmed subplots to avoid warming of control plot's soil. Soil warming started in 2005. For the current study, we added three additional plots in autumn 2007. The new plots consisted of warmed and control subplots (2 × 2 m) which were equipped with constructions for removable roofs (9 × 12 m, each). Each new (2007) plot was set 10 m aside a corresponding original (2005) plot. The 10 m buffer strip assured that roofs did not affect soil moisture in the rooting zone of trees in or near the original nonroofed plots. The new design allowed for comparing the following treatments:

- control (start 2005)
- warmed (+4 °C; start 2005)
- throughfall exclusion (temporary roof; start 2008)
- combined (warmed + temporary roof, start 2008)

Direct comparison of warmed and more recently established combined plots was feasible because CO_2_ efflux from both treatments showed similar temperature sensitivity. Warmed and combined subplots were equipped with resistance heating cables (0.4 cm diameter, TECUTE – 0.18 Ohm m^−1^ UV^−1^, Etherma, Salzburg). The cables were buried in 3 cm deep slots and had a spacing of 7–8 cm. The fully automatic warming system was controlled by a datalogger (Campbell CR 10; Campbell Scientific, Inc., North Logan, UT, USA). The soil temperature of each warmed subplot was kept 4 °C above that of the adjacent control subplot during the snow-free seasons. Soil warming during winter would have melted all snow and thereby led to unrealistic temperature and moisture conditions during winter and springtime. Temperature sensors (PT100; EMS, Brno, Czech Republic), which were used to control the warming system, were placed in the mineral soil at a depth of 5 cm half-way between two cable lines on the warmed subplots and randomly at 5 cm mineral soil depth on the control subplots. Soil warming started on May 5 in 2008, May 19 in 2009, and April 13 in 2010 and continued at least until the end of November.

**Fig. 1:**
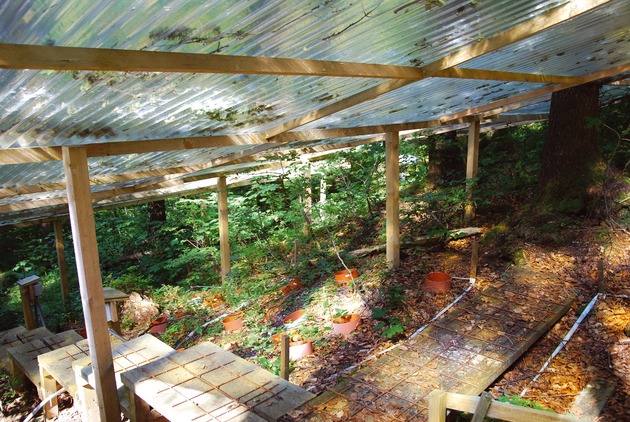
Image of one of three roof constructions in August 2009. Orange chambers were used for CO_2_ efflux measurements.

To simulate a severe summer drought, we set up three wooden roof constructions, which were covered with transparent plastic panels during 25 days in July 2008 and in July/August 2009. Each roof construction was ∼1.5 m high and had an area of ∼100 m^2^ (Fig. 1). Roofs were built around tree stems and the remaining space between stem and roof was foamed to get a tight sealing. During rain, roof water was collected with gutters and transported 50 m downhill the experimental site.

At each subplot, soil temperature was measured at 5 cm and at 15 cm mineral soil depth (PT100 temperature sensors; EMS). Soil moisture at each subplot was measured by ECH_2_O-10 soil moisture probes (Decagon, Washington, DC, USA) at 5 and 15 cm mineral soil depth. Soil temperature and moisture were measured every 20 s, and 30 min average data were stored on three data loggers (Campbell CR 10x, Campbell Scientific, Inc.; Delta-T DL2, Delta-T Devices Ltd, Cambridge, UK; MiniCube, EMS). Additionally, air temperature (ST1 sensor; Delta-T Devices Ltd) and relative air humidity (OTM-592 C sensor; Sommer, Koblach, Austria) were measured in close vicinity to the plots at 2 m height and stored as half hourly averages. Precipitation was measured with an ombrometer (NIWA/MED-K505; Sommer) at 1 min resolution (during events) at a meteorological station ∼100 m away from the site in an open area. Throughfall (precipitation reaching the soil surface in the forest stand) was estimated from 15 rain gauges (diameter 18 cm). Throughfall was collected every second week.

Soil CO_2_ fluxes were measured fortnightly during the snow-free seasons. On each subplot, soil respiration was measured from three randomly distributed chambers (20 cm diameter, 10 cm height). The chambers were inserted 1 cm into the soil to ensure an airtight seal. For flux measurements, chambers were closed with a stainless steel lid for 300 s. CO_2_ concentrations were recorded every 30 s. The CO_2_ concentration increase in the chamber headspace during the last 120 s was used to calculate the flux (linear fit). The lid had a round rubber sealing to ensure a gas tight connection to the chamber and a vent to prevent over or under-pressure in the chamber headspace. CO_2_ concentrations were measured with an EGM4 infrared gas analyzer (PP-Systems, Amesbury, MA, USA). Soil respiration measurements of all chambers took nearly 5 h. To assure a consistent measurement protocol, we started the CO_2_ flux measurements between 9:00 and 10:00 hours. The order of CO_2_ flux measurements was random, but a measurement in a control subplot was always followed by a measurement in the adjacent treatment subplot.

We further installed automated chambers to measure soil respiration at higher temporal resolution, particularly as rewetting effects on soil respiration might be overseen at fortnightly measurement intervals. We installed four automated chambers. A single chamber was installed randomly at each treatment subplot. Chambers were made of stainless steel (ø = 20 cm; Vol. = 3.27 l) and equipped with visible Plexiglass lids (with round rubber/teflon sealing), which were closed when attached magnets received power. A counterweight ensured that chambers opened again when power was cut. Lids remained at 80° inclination when chambers were open. For a more detailed description of the automated chambers, see Holtermann (1996). As automated chambers were simultaneously used for NO_x_ measurements (Kitzler *et al*., 2006), CO_2_ efflux had to be determined on an open flow-through basis. Air was sucked through openings in the chamber walls at 1.5 l min^−1^ to the analyzers (CO_2_ analyzer: WMA4; PP-Systems, Hitchin, UK). Each chamber was closed for 6 min. CO_2_ concentrations reached steady state 3–4 min after closure. Average CO_2_ data from minute 5–6 were used to calculate the flux. For that, the chamber CO_2_ concentration had to be set into relation to the CO_2_ concentration in a reference chamber. The reference chamber was similarly designed but the opening to the soil was sealed with a Plexiglas pane. The CO_2_ efflux from the soil was calculated as follows:

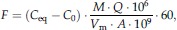
 where *F* is the net flux in mg C m^−2^ h^−1^, *M* is the atomic weight of the element (C = 12.0107 g mol^−1^), *V*_m_ is the standard gaseous molar volume (24.055 10^−3^ m^3^ mol^−1^), *C*_eq_ is the mixing ratio (ppm = 10^−6^ m^3^ m^−3^) of the gas when the chamber under investigation has reached steady state, *C*_0_ is the mixing ratio of the gas in the reference chamber, *Q* is the mass flow rate of air through the chamber (0.0015 m^3^ min^−1^), and *A* is the soil surface area of the chamber (0.0314 m^2^). Reference chamber CO_2_ concentrations were determined after every soil chamber measurement. We conducted a measurement cycle (4 soil chambers + 4 times reference chamber) every 2 h.

### Radiocarbon measurements

The radiocarbon signature of the soil CO_2_ efflux (Δ^14^C_SR_) was measured in each plot of the four treatments before (June 17), during (August 12), and after throughfall exclusion (September 22) in 2009. For the Δ^14^C_SR_ analyses, one mixed gas sample was collected from each plot (*n* = 3) per treatment by connecting the three manual soil CO_2_ efflux chambers. After closure, the headspace volume of the chambers was flushed three times with CO_2_-free synthetic air for 30 min at a flow rate of 1.5 l min^−1^. Afterwards, the chambers were sealed and incubated until the headspace reached a CO_2_ concentration of at least 1500 ppmv. Incubation time varied with the CO_2_ flux rates at the sampling day. An evacuated stainless steel sampling cylinder (2 l) was connected to the three chambers and slowly filled with gas from inside the chambers. While headspace air was sucked into the steel cylinders, a 5 l gas-bag filled with pure N_2_ was connected to the chambers to avoid under-pressure.

Additionally, three root samples were sampled at each sampling date close to the control plots for the radiocarbon signature of respired CO_2_ from live roots (Δ^14^C_RR_). Shortly after sampling, soil particles were removed and roots were cleaned with water and then separated into dead and live root fractions. Live roots were transferred into gastight containers which were flushed with CO_2_-free synthetic air to remove all atmospheric CO_2_. After an incubation time of about 1 day at a constant temperature of +15 °C, gas samples were taken using the same evacuated sampling cylinders (2 l) as described before.

*Via* mass-flow controllers the cylinders were connected to a high-vacuum extraction line at the University of Bayreuth. CO_2_ was cryogenically purified and converted to graphite targets using the modified sealed tube zinc reduction method described by Xu *et al*. (2007). Graphite targets were analyzed by the Keck Carbon Cycle AMS facility at UC Irvine, USA, with a precision of 2–3‰. Radiocarbon data are expressed as Δ^14^C, which is the per mil deviation from the ^14^C/^12^C ratio of oxalic acid standard in 1950. The sample ^14^C/^12^C ratio has been corrected to a δ^13^C value of −25‰ to account for any mass dependent fractionation effects (Stuiver & Polach, 1977).

### Microbial biomass

To assess treatment effects on soil microbial biomass, we sampled soil before, during, and after throughfall exclusion in 2008 and 2009. Mineral soil was sampled at 0–5 cm soil depth. Microbial biomass C and N were determined using a modified version of the Chloroform Fumigation Extraction method (Schinner *et al*., 1996). A detailed description of the procedure can be found in Schindlbacher *et al*. (2011) where warming effects on soil microbial biomass from the same plots were reported.

### Data analysis

Estimates of seasonal soil C losses were made by linear interpolation between sequential CO_2_ flux measurements in our time series (Sigma plot, procedure AREA). Treatment responses were analyzed with a mixed effects model with repeated measurements (sas 9.2, procedure Mixed, SAS Institute, http://www.sas.com). Treatments (control, warmed, roof, combined) and ‘date of measurement’ and the interaction (TREAT × DATE) were the fixed effects of the model, while plot was the random factor. The 3 years were analyzed separately. The homogeneity of variances was investigated with the plots of model residuals. No data transformation was necessary. For the comparison of multiple means, the Bonferroni correction was used.

## Results

### Soil temperature and moisture

**Fig. 2:**
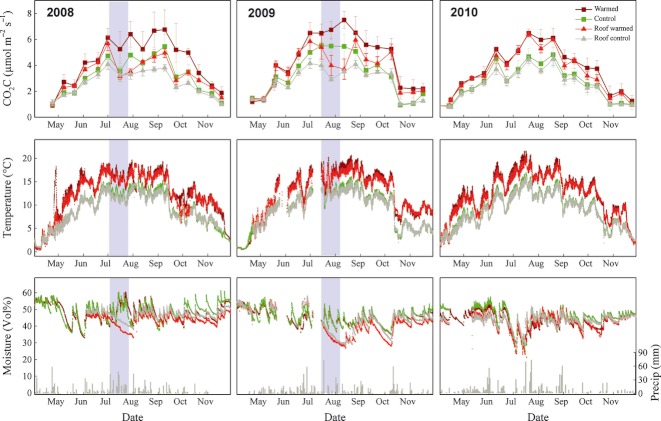
Course of soil respiration, soil temperature, soil moisture, and precipitation during snow-free seasons in 2008, 2009, and 2010. Soil respiration rates (means ± SE, *n* = 3) were determined manually at fortnightly intervals. Soil temperature and soil moisture were detected at 5 cm soil depth. Precipitation was measured in an opening ∼100 m away from the plots. Different colors represent different treatments (green: control; dark red: 4 °C soil warming; gray: throughfall exclusion; red: throughfall exclusion +4 °C soil warming). Gray shadings indicate periods when roofs were installed. No roofs were installed in 2010.

Mean soil temperatures at 5 cm soil depth of the control plots were similar with 8.83, 8.79, and 8.86 °C during the observation periods from April 1–November 30 in 2008, 2009, and 2010, respectively (Fig. 2). Artificial warming increased soil temperatures at 5 cm depth on average by 3.99 ± 0.49 °C, 3.95 ± 0.67 °C, and 3.84 ± 0.68 °C in 2008, 2009, and 2010. A short overheating of the warmed plots beneath roofs occurred in spring 2008 due to datalogger malfunction (Fig. 2). Power supply problems also caused temporary shortfall of warming between the end of September and beginning of October 2008 (Fig. 2). Soil temperature beneath installed roofs showed an almost identical course as soil temperature in plots without roofs (Fig 2; e.g., control: 12.76 ± 1.21 °C; roof-control: 12.51 ± 0.85 °C; warmed: 16.44 ± 2.13 °C; roof-warmed: 16.21 ± 1.28 °C during roof closure in 2009).

**Fig. 3:**
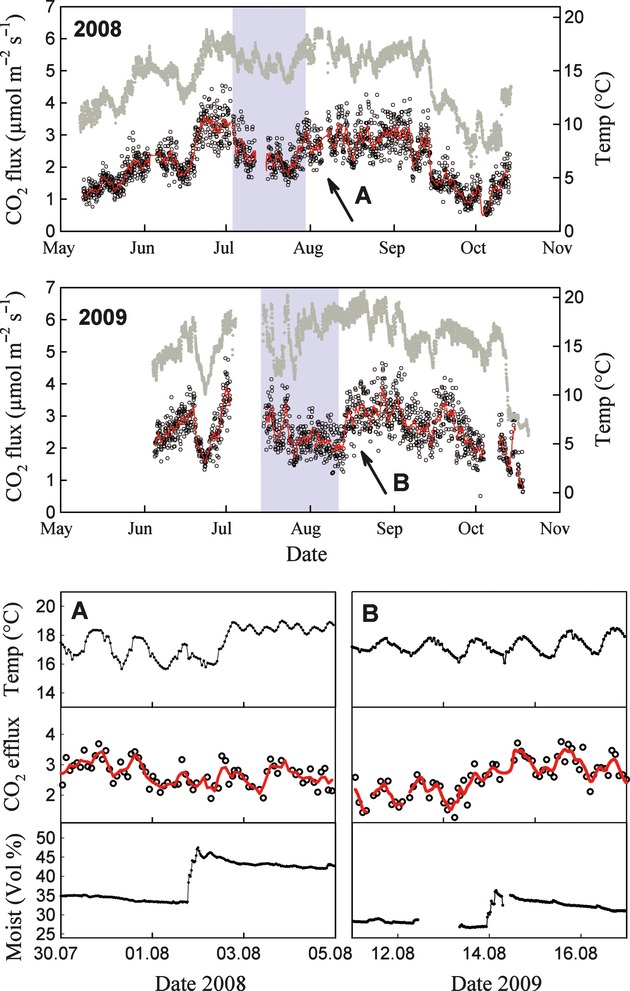
Automatically measured CO_2_ efflux (black circles; red line shows running means over 24 h) from a combined-treatment (throughfall exclusion +4 °C soil warming) plot during 2008 and 2009. Soil temperature at 5 cm soil depth is shown in gray. Gray shadings indicate periods of roof installation. The lower part of the graph shows the first rewetting phases after roof-removal in 2008 (A) and 2009 (B). Soil temperature and moisture at 5 cm soil depth, as well as soil respiration rates, are shown from 3 days prior to 3 days after first rewetting (red line: running means over 6 h).

Precipitation and soil moisture showed different patterns during the 3 years. A spring drought caused low soil moisture in 2008 (Fig. 2). In 2009, precipitation was equally distributed throughout the season, except for a dry period during late September and early October. The year 2010 was characterized by two short dry periods in June and July (Fig. 2). Annual throughfall amounted to approximately 85% of annual precipitation (Table 1). Throughfall exclusion by roofs was 16% of annual throughfall in 2008 and 12% of annual throughfall in 2009 (Table 1). Volumetric water content declined gradually under the roofs and reached lowest values of 33 and 27 vol% at 5 cm soil depth in 2008 and 2009, respectively. Soil moisture at 15 cm soil depth responded similarly, except that soil water contents were generally higher at 15 cm depth (Supporting Information, Fig. S1). At 5 and 15 cm soil depths, soil moisture decreased more pronounced in combined (warmed + roof) plots than in roof-only plots in 2008. The decrease in soil moisture was rather similar in combined and roof-only plots in 2009 (Fig. 2). The organic layer was strongly affected by throughfall exclusion, leading to almost completely dry litter and humus layers during the latter part of roof-closure. Heavy precipitation 2 days after roof-removal led to fast recovery of soil moisture in 5 cm mineral soil in 2008 (Figs 2 and 3). Less intensive precipitation after roof-removal caused slower recovery to control moisture levels in 2009 (Fig. 2). Generally, frequent precipitation kept soil moisture at high levels and raised soil moisture from warmed plots to control plot levels at short intervals, thereby preventing a drying out of the warmed soil.

**Table 1:** Annual precipitation and throughfall (precipitation reaching the forest floor) at the study site. Δ Values show precipitation and throughfall excluded during roof application. No roofs were applied in 2010

Year	Precipitation (mm)	Throughfall (mm)	Δ Precip. (mm)	Δ Throughf. (mm)

2008	1553	1313	272	210
2009	1764	1470	202	176
2010	1620	1399	–	–

### Soil CO_2_ efflux

Soil CO_2_ efflux followed the seasonal course of soil temperature in untreated-control and warmed plots in 2008, 2009, and 2010 (Fig. 2). Soil respiration rapidly responded to artificial warming, best seen in mid-May 2009, when the warming system was switched on (Fig. 2). Soil warming significantly (*P* < 0.001) increased the CO_2_ efflux from warmed and combined treatments when compared with corresponding control and roof-only treatments throughout 2008, 2009, and 2010. Warming treatment increased soil respiration (cumulative C loss during soil warming) by 32% in 2008, 36% in 2009, and 40% in 2010. Soil warming in throughfall-exclusion plots [difference between combined (warmed + roof) and roof plots] increased the cumulative CO_2_ efflux by 26% in 2008, 38% in 2009, and 42% in 2010. Warming effects in roofed plots were as persistent as in nonroofed plots, except during the latter part of roof installation, when warming effects diminished (Fig. 2).

Throughfall exclusion significantly (*P* < 0.001) decreased the soil CO_2_ efflux in 2008 and 2009. CO_2_ efflux from roof-only plots was significantly (*P* < 0.001) lower than that of all other treatments in 2008 and 2009. CO_2_ efflux from combined-treatment plots was significantly (*P* < 0.001) lower than that of warmed plots in 2008 and 2009. During the last week of roof-closure, CO_2_ efflux rates from combined-treatment plots were 45% and 51% below that of solely warmed plots in 2008 and 2009, respectively. There was no significant difference between the CO_2_ efflux from combined and untreated-control plots during the years when roofs were installed (2008: *P* = 0.40; 2009: *P* = 0.17). We did not observe a peak or even an increase in soil CO_2_ efflux during or after rewetting in 2008 (Figs 2 and 3). Soil respiration rates remained persistently low until mid-October, and albeit roofs were removed at the end of July (Fig. 2). In 2009, we observed a fast recovery of soil respiration fluxes after rewetting (Fig. 3). Shortly after roof removal, soil respiration rates recovered to control and warmed plots levels, but dropped again during the following months until October 2009 (Fig. 2).

In 2010, when no roofs were applied, CO_2_ efflux from warmed and combined-treatment plots was significantly (*P* < 0.001) higher than that of control and (formerly) roofed plots. As observed prior to roof closure in 2008 and 2009, the CO_2_ efflux from throughfall exclusion plots was slightly lower than that of the solely warmed and untreated-control plots throughout the whole 2010 season (Fig. 2, Table 2). The difference in CO_2_ efflux between warmed and combined as well as between control and (formerly) roofed plots was, however, not significant.

**Table 2:** CO_2_ efflux from different treatment plots in t C ha^−1^ (means ± SE in brackets, *n* = 3) during the observation periods in 2008, 2009, and 2010

Year	Control	Warmed	Roof	Roof + warmed

2008 (April 24–November 18)	7.14 (0.98)	9.50 (1.66)	5.89 (0.41)	7.41 (0.33)
2009 (April 21–November 17)	7.61 (0.84)	10.11 (1.31)	6.10 (0.32)	8.28 (0.98)
2010 (April 13–November 24)	6.82 (1.20)	9.35 (0.98)	6.27 (0.56)	8.72 (0.29)

### Radiocarbon

**Fig. 4:**
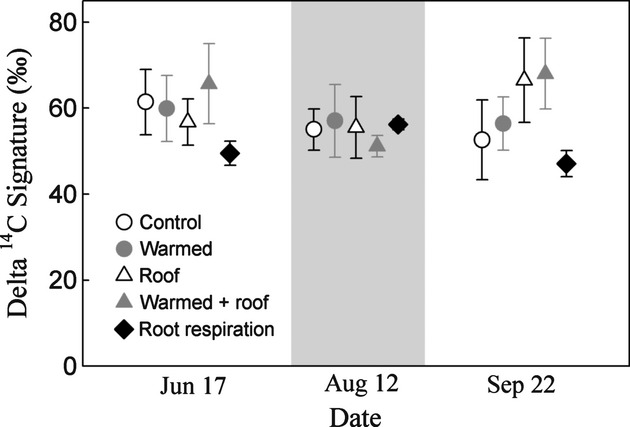
Radiocarbon signatures of soil CO_2_ efflux in the treatment plots and of CO_2_ released by incubated roots at three occasions in 2009. Gray shading indicates throughfall exclusion.

The radiocarbon signature of soil respiration varied between 51‰ and 68‰ in all treatments at the three sampling dates in 2009 (Fig. 4). The differences in the Δ^14^C among the treatments were rather small and not significant, indicating that the manipulations had generally little effect on the relative contribution of main CO_2_ sources to total soil respiration. However, although not significant, the roofed plots exhibited increasing Δ^14^C values (67‰ and 68‰) after rewetting and increased soil moisture in mid September. In the unroofed plots, mean Δ^14^C values slightly decreased from June to September.

CO_2_ released by incubated roots had radiocarbon signatures of 47–56‰. The radiocarbon signatures from the June (47‰) and September (49‰) sampling were similar to the average atmospheric CO_2_ signature (46‰) in the summer of 2009 (I. Levin, personal communication, measured at Schauinsland, Germany). Given the small differences in the Δ^14^C signature, the incubated roots respired recently fixed C. Older C was released by the roots in August 2009 when a radiocarbon signature of 56‰ was detected. On average, the difference between atmospheric and root Δ^14^C corresponded to approximately 2–3 years old C that was respired in August.

### Microbial biomass C

**Fig. 5:**
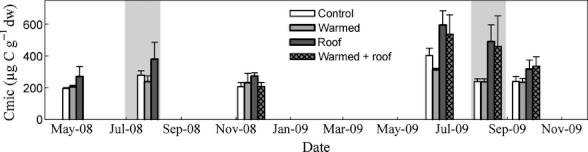
Microbial biomass C (means ± SE, *n* = 3) at 5 cm mineral soil depth from different treatment plots before, during, and after roof-closure in 2008 and 2009. During the first two dates, microbial biomass on combined-treatment (throughfall exclusion +4 °C soil warming) plots was not determined. Gray shadings indicate periods when roofs were installed.

Microbial biomass C in the throughfall-exclusion plots never decreased below microbial biomass C levels on untreated-control or warmed plots (Fig. 5). While microbial biomass C was similar in plots with and without throughfall exclusion in 2008, microbial biomass C was already higher in throughfall exclusion plots before roof application in 2009. Microbial biomass C remained at higher levels during and after throughfall reduction in 2009 (Fig. 5).

## Discussion

Our hypothesis that soil warming increased the CO_2_ efflux from the soil was confirmed by our observations. Seasonal soil CO_2_ efflux was similarly enhanced at warmed and combined-treatment plots, when compared with the corresponding control and roof-control plots. As continuous soil warming tends to reduce the amount of easily available SOM and thereby gradually decreases the warming effect (Kirschbaum, 2004; Eliasson *et al*., 2005; Hartley *et al*., 2007), we had to consider that comparison of older (2005) warmed plots with more recently established combined-treatment plots (2008) might be problematic. We observed, however, no decrease of the warming response at any plot so far. With an increase in soil CO_2_ efflux of ∼40% during 2010, the response to warming was still in the range observed at the beginning of the experiment in 2005/2006 (40–45% increase in CO_2_ efflux; Schindlbacher *et al*., 2009).

Our results suggest that this soil type responds stronger to soil warming than boreal forests soils or soils with smaller A-horizon. In such soils, the warming effect diminished already after 2–3 years (McHale *et al*., 1998; Rustad *et al*., 2001; Strömgren, 2001). Similar to our observations, prolonged warming effects were observed in a temperate mixed forest (Melillo *et al*., 2002). Their plus 5 °C soil warming treatment increased soil CO_2_ efflux throughout the first 6 years. Thereafter, the warming response leveled-off. If, how, and when the warming effect on soil CO_2_ efflux at our site will become reduced or level-off will be determined by continuing soil warming. So far, we have not observed any signs of microbial adaptations (Schindlbacher *et al*., 2011) or substrate limitation due to warming.

Effects of summer throughfall exclusion on soil CO_2_ efflux were stronger than hypothesized. The steady decrease of soil moisture during roof-closure indicated that the drought treatment functioned well. As the soil moisture record did not show any peaks during rain events (Fig. 2), lateral down-slope water flow into the soil profile did not occur or at least did not reach the plots where CO_2_ efflux was measured. Before roofs were removed soil moisture was decreased to approximately 30 vol% at 5 cm mineral soil depth during both treatment seasons. This corresponded to a pF-value of 3.6 or a water potential of −0.39 MPa (Fig. S2), representing slightly dry conditions at this soil depth. Such conditions were also observed in the control plots during naturally dry periods, but the CO_2_ efflux did not decrease as much as in the drought treatment. Naturally dry periods were always shorter than the 25 days throughfall exclusion and the forest floor was periodically rewetted at intermediate rain events. This was not the case during throughfall exclusion where the litter and humus layers gradually dried out. In 2008, the latter part of throughfall exclusion coincided with naturally dry conditions, characterized by low relative air humidity and high vapor pressure deficit (15 min average VPD over the last 7 days before roof-removal: 0.79 kPa, max.: 2.86 kPa). During this period, the forest floor fell completely dry; particularly in combined-treatment plots, where soil warming had an additive effect by reducing humidity uptake from the surrounding air. The organic layer dried out during throughfall exclusion in 2009 as well. However, VPD of surrounding air was comparatively low during throughfall exclusion in 2009 (average: 0.36 kPa, max 1.39 kPa). This should have had assured humidity uptake from air and lessened the desiccation of the organic layer to some extent. Nevertheless, also in 2009, moisture of the organic layer was far below soil moisture recorded by our sensors at 5 cm mineral soil depth. Therefore, the sharp decrease in soil CO_2_ efflux was most likely caused by drying out of the organic layer and the first centimeter topsoil during throughfall exclusion. Organic layer and topsoil are characterized by high amounts of labile SOM and highest biological activity (Schindlbacher *et al*., 2010). At our site the organic layer also contains a bigger portion of the tree fine root biomass (Díaz-Pinés *et al*., 2010). Drying of litter and organic layers was found to be the main cause of decreased CO_2_ efflux during natural drought at other forest soils as well (Scott-Denton *et al*., 2006; Cisneros-Dozal *et al*., 2007). In a similar throughfall exclusion experiment, consistently reduced matric potential in the organic layer and uppermost centimeters of the mineral horizon substantially decreased the CO_2_ efflux from a spruce forest soil in southern Germany (Muhr & Borken, 2009).

That throughfall exclusion completely dried out the organic layer might also explain why we did not observe the hypothesized peak in soil CO_2_ efflux after rewetting in 2008. Generally, rewetting of dry soil can lead to a pulse in CO_2_ efflux (Birch, 1958). Mineralization of previously unavailable, easily decomposable substrates can trigger microbial activity and cause short-term CO_2_ fluxes that even exceed predrought levels (Kieft *et al*., 1987; Borken *et al*., 2003; Wu & Brookes, 2005). Such a temporary increase in soil CO_2_ efflux may however only occur if the soil is rewetted homogenously. After long dry periods, hydrophobicity (increased water repellency) can cause water flow along preferential flow paths, bypassing hydrophobic surfaces (Ritsema & Dekker, 1996; Bogner *et al*., 2010). In coniferous forests, organic layers or part of the organic layers are often hydrophobic during drought periods (Mataix-Solera *et al*., 2007; Borken & Matzner, 2009; Muhr & Borken, 2009). Generally, the wetting resistance of soil will increase, the longer the soil is dry and the higher soil temperatures are (Goebel *et al*., 2011). Presuming that the organic layer became increasingly hydrophobic during throughfall exclusion, first precipitation events after roof-removal might have passed the organic layer without rewetting it homogenously in 2008.

Another explanation for the consistently low soil CO_2_ efflux after rewetting could be that microbial populations in the litter and organic layers were so severely affected by drought that they did not recover after rewetting. As we did not observe a decrease in microbial biomass at 0–5 cm mineral soil depth during and after drought, it seems unlikely that microbes in the organic layer above were that drastically affected. In 2009, a less dry and hydrophobic forest floor should have taken up incoming precipitation more effectively. This would explain the fast recovery of CO_2_ efflux after rewetting and the slower recovery of soil moisture in the subjacent mineral soil (Fig. 2). Comparing 2008 and 2009 and considering that we tried to simulate a summer drought, the conditions in 2008 had been more realistic, as roof installation overlapped with a period of low air humidity and high VPD.

According to the radiocarbon signature of soil CO_2_ efflux, soil warming, soil drought, and the combination of both treatments did not alter the relative contribution of single CO_2_ sources to soil CO_2_ efflux (Fig. 4). Only the increase in the radiocarbon signature following rewetting of dry soil suggests a greater contribution of heterotrophic respiration in the roofed plots. The radiocarbon signatures, however, were influenced by carbonate weathering. We cannot exclude that the weathering contributed differently to soil CO_2_ efflux in the treatment plots. Given a radiocarbon signature of −1000‰ for carbonates of the bedrock, a 1% contribution of CO_2_ from carbonate weathering would reduce the radiocarbon signature of soil CO_2_ efflux by −10‰. We assume that carbonate weathering contributed not more than 1–2% to soil CO_2_ efflux as the radiocarbon signatures were in a similar range as in other studies on carbonate-free forest soils (Muhr & Borken, 2009). Further, the treatments had probably little effect on carbonate weathering because they affected mainly the temperature and water content of the topsoil where carbonate weathering plays a minor role. If this holds true, heterotrophic and autotrophic respiration were affected to the same extent by soil warming and drying. That autotrophic and heterotrophic soil respiration was similarly affected by warming is in agreement with previous observations at our site (Schindlbacher *et al*., 2009). The effects of throughfall exclusion on autotrophic respiration could be related to the high content of fine roots in the organic layer and topsoil of the shallow soils at our site. In other studies, drought mainly affected heterotrophic soil respiration (Borken *et al*., 2006; Cisneros-Dozal *et al*., 2007; Muhr & Borken, 2009).

Contrary to our hypothesis the effect of soil warming on soil CO_2_ efflux was fully compensated in 2008 and partly compensated in 2009 by 25 days of summer throughfall exclusion (Table 2). Throughfall exclusion sharply decreased the soil CO_2_ efflux in summer, when it was naturally at highest levels. This had strong effects on the cumulative seasonal soil CO_2_ efflux. The decrease in CO_2_ efflux during roof-closure compensated only a part of the seasonal warming effects. The unexpected persistency of drought effects for several months after roof-removal was a further reason why soil warming effects were compensated. Persistently reduced soil CO_2_ efflux long after drought or a delayed impact of drought on soil CO_2_ efflux has been observed in a growing number of studies (Muhr & Borken, 2009; CLIMAITE-project: Per Ambus, personal communication). The mechanisms behind drought effects on soil CO_2_ efflux are diverse, depending on soil and humus type, species composition, current climate conditions and microbial community structure and physiology (reviewed in Borken & Matzner, 2009). Unless the underlying mechanisms are indentified, modelers are challenged in providing reliable estimates of future CO_2_ emission scenarios for forest soil. For instance, the persistency of drought effects after rewetting will not be reflected by simulation models where respiration is constrained by soil moisture. To overcome that, the assessment and incorporation of additional parameters such as soil water repellency are required (Goebel *et al*., 2011).

There is no evidence that substrate which accumulated during drought was consumed during the following years at our site. In 2010 and 2011 (A. Schindlbacher, unpublished results), CO_2_ efflux from warmed as well as control plots was similar to the CO_2_ efflux from corresponding plots which were roofed in 2008 and 2009. Hence, our results suggest that throughfall exclusion reduced the C loss from soil at our site during the year of drought.

At our temperate site, simultaneous manipulation of soil temperature and precipitation showed that warming likely causes significant C losses from the soil as long as precipitation pattern remain steady. If summer droughts become more severe (longer) in future, soil C losses by warming will likely be compensated by reduced soil CO_2_ efflux during and after drought. Our results suggest that reliable estimates of future soil C dynamics require incorporating the underlying mechanisms of SOM decomposition during and after drought into biogeochemical models.

## Supporting information

**Figure S1.** Soil moisture at 15 cm mineral soil depth during the observation periods in 2008, 2009, and 2010.

**Figure S2.** pF-curve of soil sampled between 3 and 8 cm mineral soil depth.

## References

[b1] Bellamy PH, Loveland PJ, Bradley RI, Lark RM, Kirk GJD (2005). Carbon losses from all soils across England and Wales 1978–2003. Nature.

[b2] Birch HF (1958). The effect of soil drying on humus decomposition and nitrogen availability. Plant and Soil.

[b3] Bogner C, Gaul D, Kolb A, Schmiedinger I, Huwe B (2010). Investigating flow mechanisms in a forest soil by mixed-effects modelling. European Journal of Soil Science.

[b4] Borken W, Matzner E (2009). Reappraisal of drying and wetting effects on C and N mineralization and fluxes in soils. Global Change Biology.

[b5] Borken W, Davidson EA, Savage K, Gaudinski J, Trumbore SE (2003). Drying and wetting effects on CO_2_ release from organic horizons. Soil Science Society of America Journal.

[b6] Borken W, Savage K, Davidson EA, Trumbore SE (2006). Effects of experimental drought on soil respiration and radiocarbon efflux from a temperate forest soil. Global Change Biology.

[b7] Cisneros-Dozal LM, Trumbore SE, Hanson PJ (2007). Effect of moisture on leaf litter decomposition and its contribution to soil respiration in a temperate forest. Journal of Geophysical Research.

[b8] Conant RT, Ryan MG, Ågren GI (2011). Temperature and soil organic matter decomposition rates – synthesis of current knowledge and a way forward. Global Change Biology.

[b9] Cox PM, Betts RA, Jones CD, Spall SA, Totterdell IJ (2000). Acceleration of global warming due to carbon-cycle feedbacks in a coupled climate model. Nature.

[b10] Davidson EA, Janssens IA (2006). Temperature sensitivity of soil carbon decomposition and feedbacks to climate change. Nature.

[b11] Davidson EA, Belk E, Boone RD (1998). Soil water content and temperature as independent or confounded factors controlling soil respiration in a temperate mixed hardwood forest. Global Change Biology.

[b12] Davidson EA, Trumbore SE, Amundson R (2000). Soil warming and organic carbon content. Nature.

[b13] Davidson EA, Janssens IA, Luo Y (2005). On the variability of respiration in terrestrial ecosystems: moving beyond Q_10_. Global Change Biology.

[b14] Díaz-Pinés E, Schindlbacher A, Pfeffer M, Jandl R, Zechmeister-Boltenstern S, Rubio A (2010). Root trenching - a useful tool to estimate autotrophic soil respiration? Case study in an Austrian mountain forest. European Journal of Forest Research.

[b15] Eliasson PE, Mcmurtie RE, Pepper DA, Strömgren M, Linder S, Ågren GI (2005). The response of heterotrophic CO_2_ flux to soil warming. Global Change Biology.

[b16] Epron D, Farque L, Lucot E, Badot PM (1999). Soil CO_2_ efflux in a beech forest: the contribution of root respiration. Annals of Forest Science.

[b17] Friedlingstein P, Cox P, Betts R (2006). Climate-carbon cycle feedback analysis: results from the C4MIP model intercomparison. Journal of Climate.

[b18] Gaumont-Guay D, Black TA, Griffis TJ, Barr AG, Jassal RS, Nesic Z (2006). Interpreting the temperature dependence of soil respiration on soil temperature and water content in a boreal aspen stand. Agricultural and Forest Meteorology.

[b19] Goebel MO, Bachmann J, Reichstein M, Janssens IA, Guggenberger G (2011). Soil water repellency and its implications for organic matter decomposition – is there a link to extreme climatic events?. Global Change Biology.

[b20] Gu L, Post WM, King AW (2004). Fast labile carbon turnover obscures sensitivity of heterotrophic respiration from soil to temperature: a model analysis. Global Biogeochemical Cycles.

[b21] Hartley IP, Heinemeyer A, Ineson P (2007). Effects of three years of soil warming and shading on the rate of soil respiration: substrate availability and not thermal acclimation mediates observed response. Global Change Biology.

[b22] Högberg P, Read DJ (2006). Towards a more plant physiological perspective on soil ecology. Trends in Ecology and Evolution.

[b23] Holtermann C (1996). A transportable system for the on-line-measurement of NO_x_ (NO, NO_2_)-emission from soils. Die Bodenkultur.

[b24] IPCC (2007). Climate Change 2007: The Physical Science Basis. Contribution of Working Group I to the Fourth Assessment Report of the Intergovernmental Panel on Climate Change.

[b25] Jassal RS, Black TA, Novak MD, Gaumont-Guay D, Nesic Z (2008). Effect of soil water stress on soil respiration and its temperature sensitivity in an 18-year-old temperate Douglas-fir stand. Global Change Biology.

[b26] Jones CD, Cox PM, Huntingford C (2003). Uncertainty in climate-carbon-cycle projections associated with the sensitivity of soil respiration to temperature. Tellus.

[b27] Karhu K, Fritze H, Hämäläinen K (2010). Temperature sensitivity of soil carbon fractions in boreal forest soil. Ecology.

[b28] Kieft TL, Soroker E, Firestone MK (1987). Microbial biomass response to rapid increase in water potential when dry soil is wetted. Soil Biology & Biochemistry.

[b29] Kirschbaum MUF (2004). Soil respiration under prolonged soil warming: are rate reductions caused by acclimation or substrate loss?. Global Change Biology.

[b30] Kitzler B, Zechmeister-Boltenstern S, Holtermann C, Skiba U, Butterbach-Bahl K (2006). Controls over N_2_O, NO_x_ and CO_2_ fluxes in a calcareous mountain forest soil. Biogeosciences.

[b31] Loibl W, Beck A, Dorninger M,, Formayer H, Gobiet A, Schöner W (2007). Kwiss-Programm reclip:more: research for climate protection:model run evaluation. Final Report, ARC-sys-0123. Austrian Research Centers–systems research, Vienna. http://systemsresearch.arcs.ac.at/SE/projects/reclip/.

[b32] Lavigne MB, Foster RJ, Goodine G (2004). Seasonal and annual changes in soil respiration in relation to soil temperature, water potential and trenching. Tree Physiology.

[b33] Luo Y, Wan S, Hui D, Wallace LL (2001). Acclimatization of soil respiration to warming in a tall grass prairie. Nature.

[b34] Mataix-Solera J, Arcenegui V, Guerrero C (2007). Water repellency under different plant species in a calcareous forest soil in a semiarid Mediterranean environment. Hydrological Processes.

[b35] McHale PJ, Mitchell MJ, Bowles FP (1998). Soil warming in a northern hardwood forest: trace gas fluxes and leaf litter decomposition. Canadian Journal of Forest Research.

[b36] Melillo JM, Steudler PA, Aber JD (2002). Soil warming and carbon-cycle feedbacks to the climate system. Science.

[b37] Muhr J, Borken W (2009). Delayed recovery of soil respiration after wetting of dry soil further reduces C losses from a Norway spruce forest soil. Journal of Geophysical Research.

[b38] Orchard VA, Cook FJ (1983). Relationship between soil respiration and soil moisture. Soil Biology & Biochemistry.

[b39] Reichstein M, Rey A, Freibauer A (2003). Modelling temporal and large-scale spatial variability of soil respiration from soil water availability, temperature and vegetation productivity indices. Global Biogeochemical Cycles.

[b40] Risk D, Kellman L, Beltrami H, Diochon A (2008). In situ incubations highlight the environmental constraints on soil organic carbon decomposition. Environmental Research Letters.

[b41] Ritsema CJ, Dekker LW (1996). Water repellency and its role in forming preferred flow paths in soils. Australian Journal of Soil Research.

[b42] Rustad LE, Fernandez I (1998). Experimental soil warming effects on CO_2_ and CH_4_ flux from a low elevation spruce-fir forest soil in Maine, USA. Global Change Biology.

[b43] Rustad LE, Campbell JL, Marion GM (2001). A meta-analysis of the response of soil respiration, net nitrogen mineralization, and aboveground plant growth to experimental ecosystem warming. Oecologia.

[b44] Schimel J, Balser TC, Wallenstein MD (2007). Microbial stress response physiology and its implications for ecosystem function. Ecology.

[b45] Schindlbacher A, Zechmeister-Boltenstern S, Jandl R (2009). Carbon losses due to soil warming: Do autotrophic and heterotrophic soil respiration respond equally?. Global Change Biology.

[b46] Schindlbacher A, De Gonzalo C, Díaz-Pinés E (2010). Temperature sensitivity of forest soil organic matter decomposition along two elevation gradients. Journal of Geophysical Research.

[b47] Schindlbacher A, Rodler A, Kuffner M, Kitzler B, Sessitsch A, Zechmeister-Boltenstern S (2011). Experimental warming effects on the microbial community of a temperate mountain forest soil. Soil Biology & Biochemistry.

[b48] Schinner F, Öhlinger T, Kandeler E, Margesin R (1996). Methods in Soil Biology.

[b49] Scott-Denton LE, Rosenstiel TN, Monson RK (2006). Differential controls by climate and substrate over the heterotrophic and rhizospheric components of soil respiration. Global Change Biology.

[b50] Stark J, Firestone MK (1995). Mechanisms for soil moisture effects on activity of nitrifying bacteria. Applied and Environmental Microbiology.

[b51] Strömgren M (2001). Soil-Surface CO_2_ Flux and Growth in a Boreal Norway Spruce Stand: Effects of Soil Warming and Nutrition.

[b52] Stuiver M, Polach HA (1977). Discussion: reporting of ^14^C data. Radiocarbon.

[b53] Trumbore SE, Chadwick OA, Amundson R (1996). Rapid exchange between soil carbon and atmospheric carbon dioxide driven by temperature change. Science.

[b54] Wu J, Brookes PC (2005). The proportional mineralisation of microbial biomass and organic matter caused by air-drying and rewetting of grassland soil. Soil Biology & Biochemistry.

[b55] Xu X, Trumbore SE, Zheng S, Southon JR, Mcduffee KE, Luttgen M, Liu JC (2007). Modifying a sealed tube zinc reduction method for preparation of AMS graphite targets: reducing background and attaining high precision. Nuclear Instruments and Methods in Physical Research, Section B.

[b56] Yuste JC, Janssens IA, Carrara A, Meiresonne L, Ceulemans R (2003). Interactive effects of temperature and precipitation on soil respiration in a temperate maritime pine forest. Tree Physiology.

[b57] Yuste JC, Baldocchi DD, Gershenson A, Goldstein A, Mission L, Wong S (2007). Microbial soil respiration and its dependency on carbon inputs, soil temperature and moisture. Global Change Biology.

