# Supplementary figures and images for: Soil respiration under climate change: prolonged summer drought offsets soil warming effects
Source: Glob Chang Biol. 2012 Apr 24;18(7):2270–9. doi: 10.1111/j.1365-2486.2012.02696.x (PMC3602719; doi:10.1111/j.1365-2486.2012.02696.x)

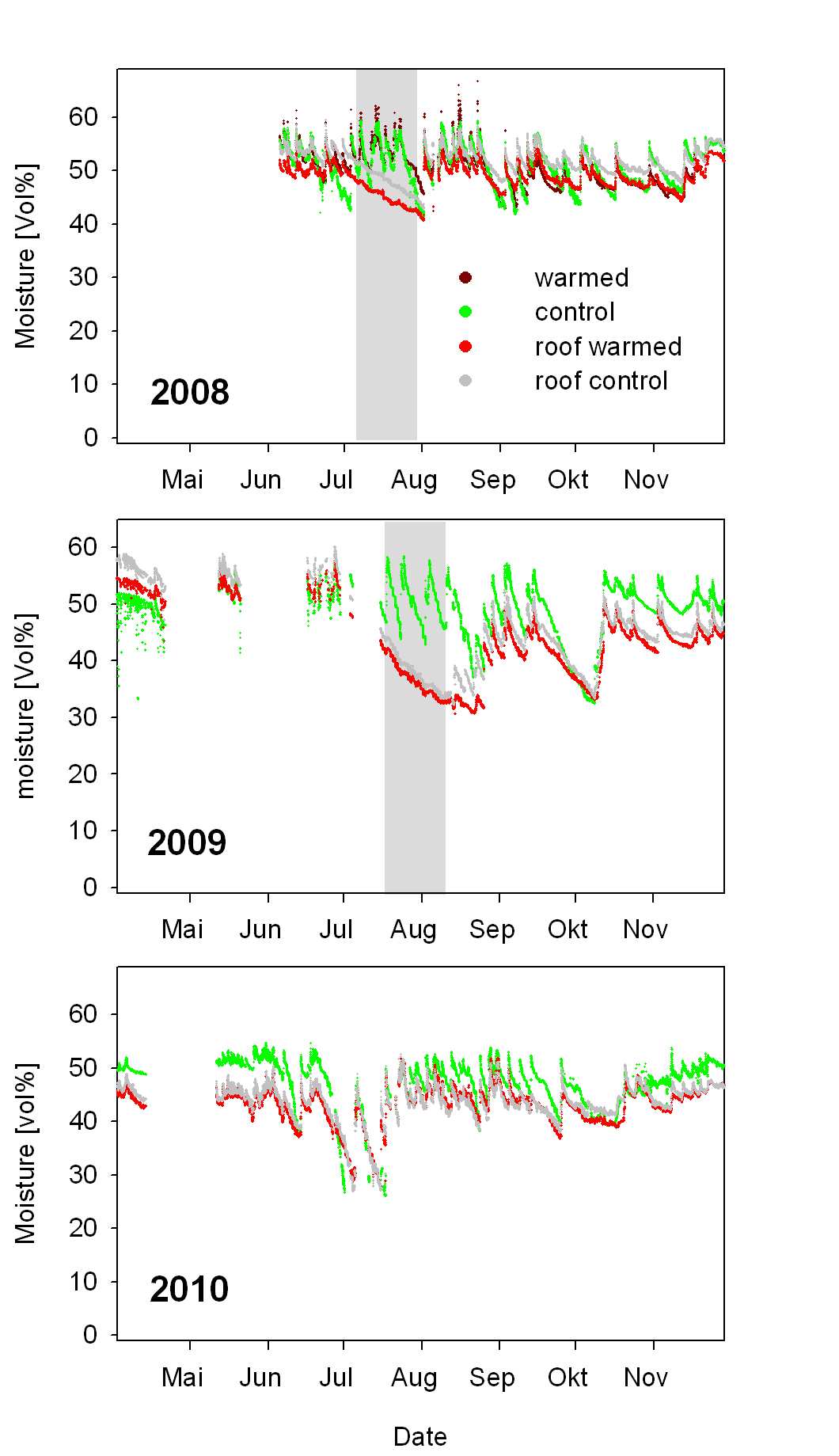

Supplement: Supplementary file 1 [file gcb0018-2270-SD1.jpg]

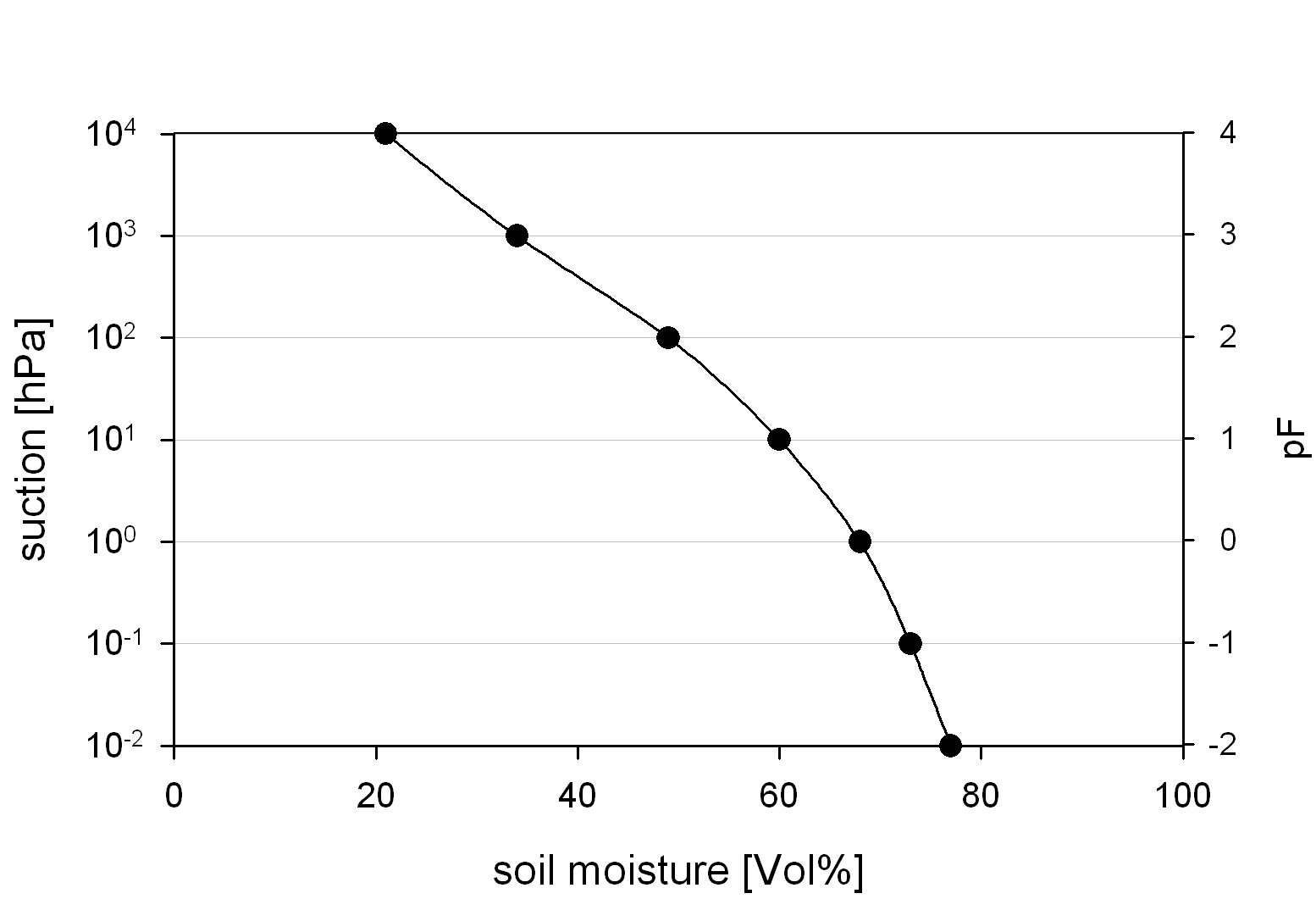

Supplement: Supplementary file 2 [file gcb0018-2270-SD2.jpg]
